# Supplementary material for: Identification of photoperiod-induced specific miRNAs in the adrenal glands of Sunite sheep (Ovis aries)
Source: Front Vet Sci. 2022 Jul 22;9:888207. doi: 10.3389/fvets.2022.888207 (PMC9354845; doi:10.3389/fvets.2022.888207)
Supplement: Supplementary file 1 [file Data_Sheet_1.ZIP › Supplementary file/miRDeep2 file.zip!/miRDeep2 file/Know_Structure/SP42A1/oar-mir-127.pdf]

miRBase precursor : oar-mir-127
Total read count : 75717
oar-miR-127 read count : 74226
remaining reads : 1491

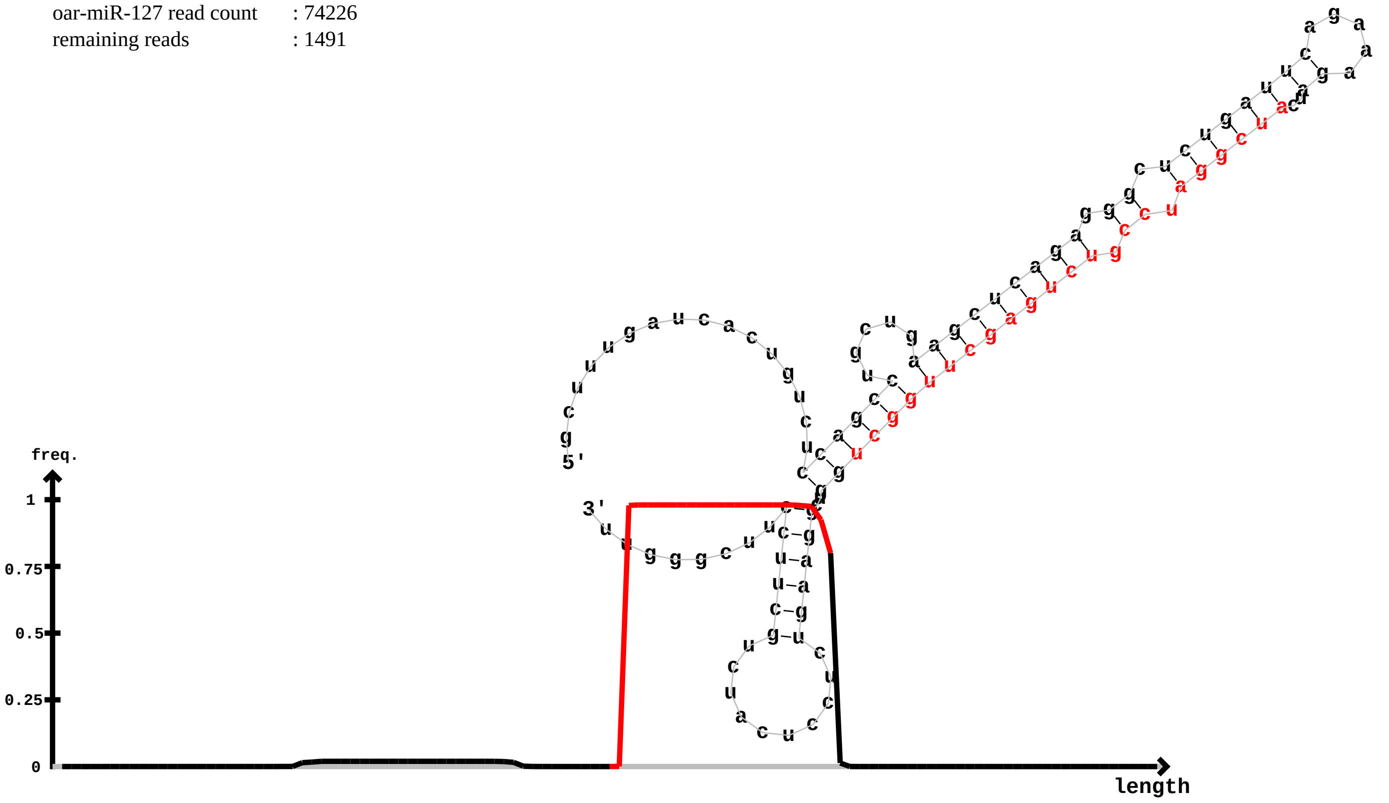

oar-miR-127

5'- gcuuugaucacugucuccagccugcugaagcucagagggcucugauucagaaagaucaucggauccgucugagcuuggcuggucggaagucuccucaucugcuuccuucgggguu -3'	exp		
...............(((((.....(((((((((.((.((((((((.....))..)))))).)).))))))))))))))..((((((..........)))))).............	reads	mm	sample
.cuuugaucacugucuccagccug..	1	0	seq
..uuugaucacugucuccagccug..	1	0	seq
....ugaucacugucuccagccug..	2	0	seq
......................ugcugaagcucagagggcucug..	3	0	seq
......................ugcugaagcucagagggcucuga...	9	0	seq
......................ugcugaagcucagagggcucugau..	4	0	seq
......................ugcugaagcucagagggcucugauu...	1	0	seq
.......................gcugaagcucagagggcucuga...	3	0	seq
.......................gcugaagcucagagggcucugau..	3	0	seq
.......................gcugaagcucagagggcucugauu...	1	0	seq
........................cugaagcucagagggcuU..	1	1	seq
........................cugaagcucagagggcuc..	1	0	seq
........................cugaagcucagagggcucu...	2	0	seq
........................cugaagcucagagggcucug..	5	0	seq
........................cugaagcucagagggcucuga...	45	0	seq
........................cugaagcucagagggcucugau..	209	0	seq
........................cugaagcucagagggcucugauu...	788	0	seq
........................cugaagcucagagggcucugauuc..	23	0	seq
........................cugaagcucagagggcucugauuca...	1	0	seq
........................cugaagcucagagggcucugauucagaa..	1	0	seq
.........................ugaagcucagagggcucuga...	1	0	seq
.........................ugaagcucagagggcucugau..	9	0	seq
.........................ugaagcucagagggcucugauu...	149	0	seq
.........................ugaagcucagagggcucugauuc..	3	0	seq
.........................ugaagcucagagggcucugauucagaaagauc...	1	0	seq
..........................gaagcucagagggcucugau..	3	0	seq
..........................gaagcucagagggcucugauu...	130	0	seq
..........................gaagcucagagggcucugauuc..	70	0	seq
..........................gaagcucagagggcucugauuca...	6	0	seq
..........................gaagcucagagggcucugauucag..	1	0	seq
...........................aagcucagagggcucugauu...	10	0	seq
...........................aagcucagagggcucugauuc..	1	0	seq
...........................aagcucagagggcucugauucag..	3	0	seq
....ucaucggauccgucugagcuugg......................................	1	0	seq
....caucggauccgucugagcuuggc.....................................	1	0	seq

oar-miR-127

```
gcuuugaucacugucuccagccugcugaagcucagagggcucugauucagaaagaucaucggauccgucugagcuuggcuggucggaagucuccucaucugcuuccuucggguu
.........................................................aucggauccgucugagcuu.......................................    2       0       seq
.........................................................aucggauccgucugagcuugg.....................................    11      0       seq
.........................................................aucggauccgucugagcuuggc....................................    27      0       seq
.........................................................aucggauccgucugagcuuggcu...................................    5       0       seq
..........................................................ucggauccgucugagcu........................................    5       0       seq
..........................................................ucggauccgucugagcuu.......................................    176     0       seq
..........................................................ucggauccgucugagcuug......................................    230     0       seq
..........................................................ucggauccgucugagcuugg.....................................    3787    0       seq
..........................................................ucggauccgucugagcuuggc....................................    9404    0       seq
..........................................................ucggauccgucugagcuuggcu...................................    59453   0       seq
..........................................................ucggauccgucugagcuuggcug..................................    982     0       seq
..........................................................ucggauccgucugagcuuggcugg.................................    11      0       seq
..........................................................ucggauccgucugagcuuggcuggu................................    1       0       seq
...........................................................cggauccgucugagcuu.......................................    2       0       seq
...........................................................cggauccgucugagcuug......................................    2       0       seq
...........................................................cggauccgucugagcuugg.....................................    5       0       seq
...........................................................cggauccgucugagcuuggc....................................    10      0       seq
...........................................................cggauccgucugagcuuggcu...................................    93      0       seq
...........................................................cggauccgucugagcuuggcug..................................    9       0       seq
...........................................................cggauccgucugagcuuggcugg.................................    1       0       seq
............................................................ggauccgucugagcuugg.....................................    1       0       seq
............................................................ggauccgucugagcuuggcu...................................    7       0       seq
```
